# Prevalence of MRI findings in adults with scapular pain: An exploratory cross-sectional study

**DOI:** 10.1016/j.inpm.2026.100827

**Published:** 2026-10-16

**Authors:** Chetan K. Potu, Henry J. Wong, Joshua Levin, Raymond Chou

**Affiliations:** a Division of Physical Medicine & Rehabilitation, Department of Orthopaedic Surgery, Stanford University School of Medicine, Redwood City, CA, USA; b Department of Orthopaedic Surgery, Stanford University School of Medicine, Redwood City, CA, USA

**Keywords:** Scapular pain, Cervical spondylosis, Supraspinatus tendinopathy, Magnetic resonance imaging, Musculoskeletal imaging

## Abstract

**Background:**

Scapular pain has multiple possible pain generators including cervical radiculopathy, facet arthropathy, scapular dyskinesis, and myofascial pain making selecting an appropriate interventional target challenging. Magnetic resonance imaging (MRI) may be ordered as part of the workup, but the prevalence of imaging findings has not been reported.

**Objective:**

To explore the prevalence of findings identified on MRI in patients with scapular pain.

**Methods:**

This single-center exploratory cross-sectional study included adults presenting to orthopedic clinics from June 2015-June 2025 with scapular pain. Prevalence of MRI findings in the cervicothoracic spine, shoulder, and scapula were determined.

**Results:**

284 of 503 patients (56%) obtained at least one MRI: cervical spine (203), thoracic spine (76), shoulder (106), and scapula (22). The most prevalent findings were supraspinatus tendinopathy (88%), C5-6 foraminal stenosis (66%), and C5-6 degenerative changes (65%).

**Conclusions:**

C5-6 pathology and supraspinatus tendinopathy were the most prevalent findings in patients with scapular pain. Incidental MRI findings in the spine and shoulder increase with age, and therefore MRI findings in patients with scapular pain should be cautiously interpreted within appropriate clinical context.

## 1 Introduction

Scapular pain, located along the superomedial scapular border near the cervicothoracic spine, often poses a diagnostic challenge [1]. Pain in this region has been reported to occur from cervical radiculopathy [2], [3], cervical and upper thoracic facet arthropathy [4], [5], [6], scapular dyskinesis [7], [8], and myofascial pain from the trapezius, levator scapulae, and/or rhomboid major [1]. Less commonly, pain can also be referred to this region from pulmonary masses [9]. Because there are multiple possible sources of scapular pain, precise identification of the primary source is necessary for selecting an appropriate intervention.

Clinical history taking and physical examination are often utilized to determine the etiology of scapular pain. Scapular pain secondary to cervical radiculopathy classically co-occurs with neck pain and arm weakness and/or paresthesia in a myotomal/dermatomal pattern of the affected nerve root [3]. Scapular pain secondary to shoulder injury includes rotator cuff disease and labral tears and may have physical examination findings of a positive Neer's sign, Hawkin's test, or O'Brien's test [10], [11].

In cases of isolated scapular pain, the source of pain may be more difficult to determine from clinical history and physical examination. In these cases, clinicians may obtain magnetic resonance imaging (MRI) of the scapula, cervicothoracic spine, or shoulder for further workup when pain does not improve with time and physical therapy. However, it has not previously been studied 1) when clinicians decide to order MRI in patients with scapular pain, 2) which MRIs are ordered, and 3) what imaging abnormalities are commonly identified in patients with scapular pain. The goal of this exploratory study was to identify the prevalence of imaging findings in patients with scapular pain.

## 2 Methods

### 2.1 Study design

This was a single-center exploratory cross-sectional study of patients who are age 18 or older who presented with scapular pain to an outpatient orthopedic clinic network (Stanford Healthcare, Stanford, CA) between June 1, 2015 and June 1, 2025. The study was approved by the Institutional Review Board at Stanford University (Protocol #81567) with a waiver of informed consent due to the retrospective study design and was conducted in accordance with the Strengthening the Reporting of Observational Studies in Epidemiology (STROBE) guidelines.

Patient cases were identified using the Stanford Research Repository (STARR) using the search term “scapula(r) pain,” and demographic information (age and sex), clinic visit notes confirming scapular pain, and MRI reports by board-certified radiologists of the cervical spine, thoracic spine, shoulder, and scapula were extracted. Cases were further analyzed if they had at least one MRI of either the cervical spine, thoracic spine, shoulder, or scapula following their initial orthopedic clinic visit for scapular pain. For cases where there were multiple MRIs performed of the same anatomic region, only the first MRI of that anatomic region after the initial orthopedic clinic visit was included for analysis.

Two physical medicine and rehabilitation physicians (C.*P. and* R.C.) reviewed the MRI reports and coded for the presence or absence of reported findings as categorized in Table 1. Cervical and thoracic spine findings were further coded by laterality (left, right, bilateral, or unspecified) and spinal level. Findings that were not reported as present or absent in the MRI reports and laterality that was not specified were treated as missing data. Any discrepancies in coding between the two reviewers were reconciled through regularly scheduled meetings.

**Table 1:** Keywords in radiology reports used to code presence of imaging findings.

	Coded Categories	Examples
**Cervical Spine**	Degenerative changes	“Disc osteophyte complex”
		“Uncovertebral hypertrophy”
		“Facet hypertrophy”
	Neural foraminal stenosis	“Foraminal narrowing”
**Thoracic Spine**	Degenerative changes	“Disc osteophyte complex”
		“Degenerative change”
	Neural foraminal stenosis	“Foraminal narrowing”
**Shoulder**	Glenohumeral joint degenerative changes	“Joint effusion”
		“Chondrosis”
		“Chondral thinning”
	Acromioclavicular joint degenerative changes	“Degenerative changes”
		“Capsular hypertrophy”
		“Distal clavicle spurring”
		“Joint effusion”
	Supraspinatus tendinopathy	“Tendinopathy”
		“Tendinosis”
		“Tendinitis”
		“Tear”
	Subacromial bursitis	“Bursal fluid”
	Labral injury	“Tear”
		“Labral cyst”
**Scapula**	Scapulothoracic bursitis	“Bursal fluid”
	Periscapular muscle changes	“Muscular atrophy or edema”

### 2.2 Statistical analysis

Statistical analyses were performed using Python (version 3.12.4; Python Software Foundation). For this exploratory study, two-sided p-value of less than 0.05 was considered statistically significant. Demographics between those with an MRI and those without an MRI were compared using chi-squared for categorical variables and one-way ANOVA for continuous variables. Demographics between those with imaging findings present and absent were compared using Fisher's exact tests for categorical variables and two-sample t-tests for continuous variables.

## 3 Results

### 3.1 Baseline characteristics

A total of 503 unique patient cases of scapular pain were identified, and 284 received at least one MRI over the ten-year study time period (56%) (Fig. 1). On the initial orthopedic clinic visit, 60% of patients were provided with a suspected anatomically localized etiology for their scapular pain, most frequently suspected to originate from the cervical spine (46%), followed by the shoulder (8%), thoracic spine (4%), and scapula (2%). There was no significant difference in the age, sex, laterality of scapular pain, or suspected diagnosis between patients who did and did not receive an MRI (Table 2).

**Fig. 1:**
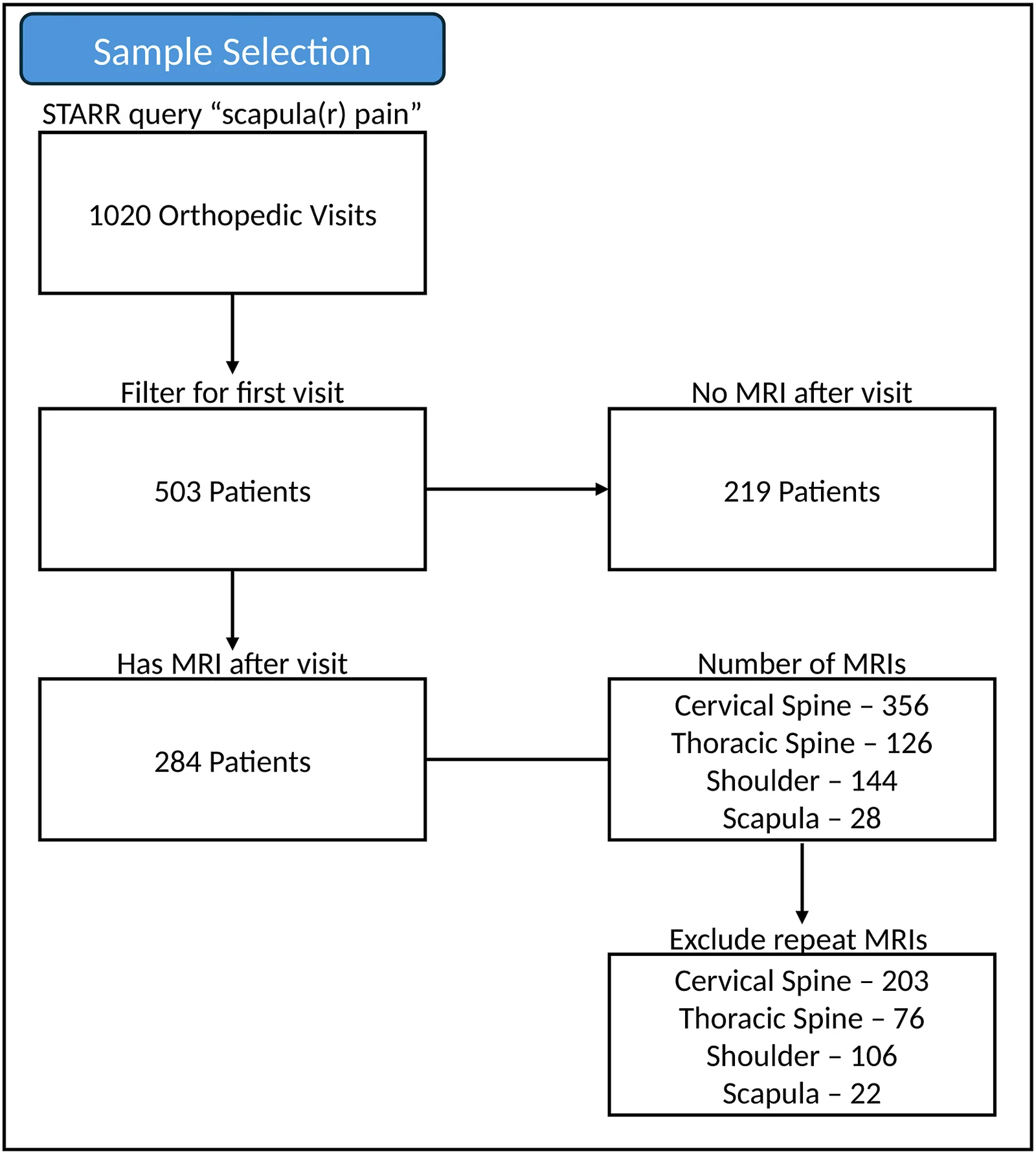
**Legend:** Flow diagram of study sample selection.

**Table 2:** Sample demographics stratified by presence/absence of MRI.

	MRI (n = 284)	No MRI (n = 219)	P-value
**Age (mean, SD)**	51 (17)	50 (15)	0.43
**Biological Sex (% female)**	151 (53%)	104 (47%)	0.21
**Laterality (%)**			0.48
**Left**	133 (47%)	97 (44%)	
**Right**	109 (38%)	95 (43%)	
**Bilateral**	42 (15%)	27 (12%)	
**Visit Clinical Diagnosis (%)**^a^			
**C spine pathology**	115 (51%)	116 (59%)	0.14
**T spine pathology**	10 (4%)	11 (6%)	0.66
**Shoulder pathology**	23 (10%)	15 (8%)	0.40
**Scapula pathology**	4 (2%)	6 (3%)	0.53

a Percentages are based on n = 225 in the MRI group and 198 in the No MRI group due to missing data; MRI = magnetic resonance imaging, SD = standard deviation.

203 patients received at least one MRI of the cervical spine (40%), 76 of the thoracic spine (15%), 106 of the shoulder (21%), and 22 of the scapula (4%).

### 3.2 Time to imaging

The median time to first MRI was 8 months (IQR: 1-31 months, range 0-130 months). MRI of the cervical spine was most frequently ordered first (60% of first MRIs), and 28 patients had multiple anatomic regions imaged for their first MRI (14% of patients who received MRIs). 68 additional patients (24% of those who received MRIs) received another MRI of a different region at a second time point, with median time of 10 months between MRIs (IQR: 3-29 months, range 0.1-112 months).

### 3.3 Imaging findings

The most common MRI findings were supraspinatus tendinopathy (88%), C5-6 neural foraminal stenosis (66%), and C5-6 degenerative changes (65%) (Table 3, Table 4). The least common findings were scapulothoracic bursitis (0%) and thoracic spine degenerative changes (0-7%). All three of the most common MRI findings were associated with age (p < 0.01) but not sex (Fig. 2). The laterality of cervical or thoracic spine findings at any level was not associated with the laterality of scapular pain. For the shoulder and scapula, association of laterality was not performed as significantly more patients received MRIs of the symptomatic side (shoulder: 82%; scapula: 100%) than the asymptomatic side (shoulder: 27%; scapula: 0%) (p < 0.001).

**Table 3:** Prevalence of MRI findings in the cervicothoracic spine by level and pathology laterality.

	Degenerative Changes				Neural Foraminal Stenosis			
	Total	Bilateral	Left	Right	Total	Bilateral	Left	Right
C2-3	42 (26%)	25 (16%)	13 (8%)	4 (3%)	29 (15%)	8 (4%)	16 (8%)	5 (3%)
C3-4	67 (48%)	47 (34%)	11 (8%)	9 (6%)	80 (41%)	41 (21%)	20 (10%)	19 (10%)
C4-5	72 (54%)	62 (47%)	5 (4%)	5 (4%)	95 (48%)	61 (31%)	24 (12%)	10 (5%)
C5-6	79 (65%)	69 (57%)	7 (6%)	3 (3%)	128 (66%)	91 (47%)	25 (13%)	12 (6%)
C6-7	73 (58%)	58 (46%)	10 (8%)	5 (4%)	110 (56%)	71 (36%)	26 (13%)	13 (7%)
C7-T1	25 (27%)	23 (15%)	2 (1%)	1 (1%)	22 (11%)	12 (6%)	6 (3%)	4 (2%)
T1-2	1 (4%)	1 (4%)	0 (0%)	0 (0%)	4 (7%)	4 (7%)	0 (0%)	0 (0%)
T2-3	1 (4%)	0 (0%)	1 (4%)	0 (0%)	6 (11%)	3 (5%)	2 (3%)	1 (2%)
T3-4	0 (0%)	0 (0%)	0 (0%)	0 (0%)	3 (5%)	2 (3%)	1 (2%)	0 (0%)
T4-5	0 (0%)	0 (0%)	0 (0%)	0 (0%)	3 (5%)	2 (3%)	1 (2%)	0 (0%)
T5-6	0 (0%)	0 (0%)	0 (0%)	0 (0%)	4 (7%)	1 (2%)	2 (3%)	1 (2%)
T6-7	0 (0%)	0 (0%)	0 (0%)	0 (0%)	3 (5%)	2 (3%)	1 (2%)	0 (0%)
T7-8	1 (4%)	0 (0%)	0 (0%)	1 (4%)	0 (0%)	0 (0%)	0 (0%)	0 (0%)

^a^Percentages are based on n = 203 for spinal levels C2-T1 and n = 76 for spinal levels T1-8.

**Table 4:** Prevalence of MRI findings in the shoulder and scapula.

MRI Finding	Total
*Glenohumeral Joint Degenerative Changes*	40 (38%)
*Acromioclavicular Joint Degenerative Changes*	62 (59%)
*Supraspinatus Tendinopathy*	93 (88%)
*Subacromial Bursitis*	52 (49%)
*Labral Injury*	45 (42%)
*Scapulothoracic Bursitis*	0 (0%)
*Periscapular Muscle Edema*	6 (27%)

^a^Percentages are based on n = 106 for the shoulder and n = 22 for the scapula.

**Fig. 2:**
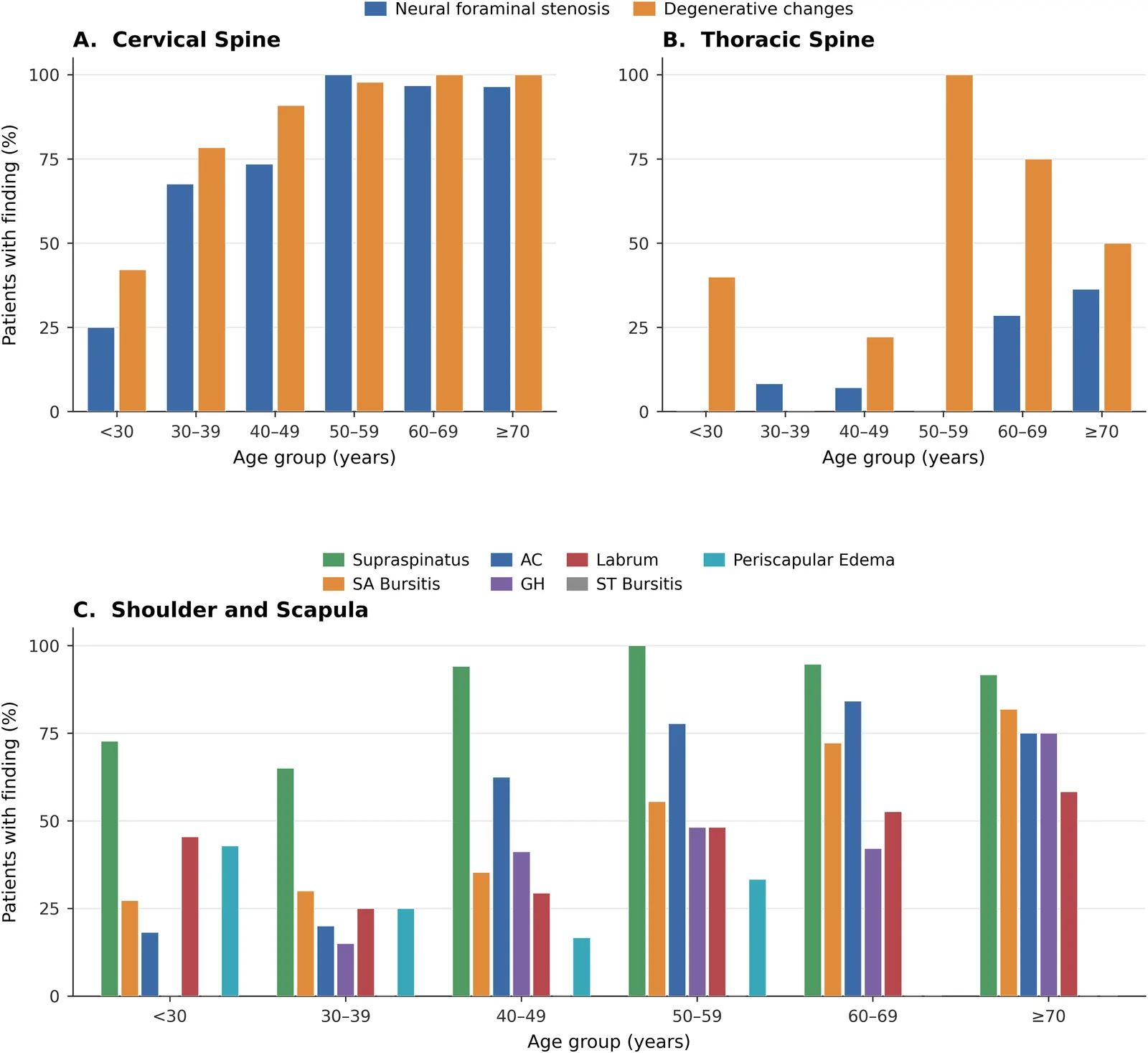
**Legend:** Prevalence of MRI findings by age decade ^a^MRI = magnetic resonance imaging, GH = glenohumeral, AC = acromioclavicular, SA = Subacromial, ST = scapulothoracic.

## 4 Discussion

### 4.1 Imaging utilization

Our study findings demonstrate the difficulty that clinicians face with diagnosing the etiology of scapular pain [2], [3], [7], [9], [12]. Only 60% of patients were provided with a suspected anatomically localized etiology for their pain after their initial clinic visit with an orthopedic specialist. While scapular pain was most often suspected to be due to cervical spine findings, 14% of patients had multiple anatomic regions imaged simultaneously, suggesting diagnostic uncertainty. An additional 24% received a second MRI at a later time point. While the reason for additional imaging was not assessed in the present study, it is possible that the imaging findings from the first MRI did not sufficiently explain the etiology of the scapular pain, or that treatment of that anatomic region did not improve the scapular pain. These results support the need for more research on the most appropriate diagnostic workup for patients with scapular pain.

### 4.2 Imaging prevalence

Supraspinatus tendinopathy (88%), C5-6 spondylosis (65%), and C5-6 foraminal stenosis (66%) were identified as the most prevalent MRI findings in patients with scapular pain. However, the presence of the abnormalities in this study were shown to be significantly associated with age. This supports the findings of previous studies that demonstrated the prevalence of supraspinatus tendinopathy and rotator cuff disease prevalence increase with age [13], [14], [15], [16], [17], and that C5-6 degenerative changes are present in over 30% of individuals by the third decade and over 80% by the ninth [18], [19], [20]. Thus, future prospective studies on scapular pain may need to control for age as a possible confounder.

The least prevalent MRI findings in this study were scapulothoracic bursitis (0%) and thoracic spine degenerative changes (0-7%). While scapulothoracic bursitis has been theorized as a source of scapular pain due to friction of the scapula sliding over the thoracic ribcage, our study results agree with prior studies that similarly have shown this is rarely seen on imaging [21], [22].

The present study did not find an association between the laterality of scapular pain and the laterality of cervicothoracic spine findings. This may be due to the high prevalence of bilateral findings in the spine. For example, 87% of patients with C5-6 degenerative changes and 71% of patients with C5-6 foraminal stenosis on MRI had bilateral findings. Though prior studies have shown that provocation of cervical and thoracic facet joints can cause scapular pain [4], [5], [6], and cervical nerve root blocks can reduce scapular pain in patients with cervical radiculopathy [3], the high prevalence of bilateral imaging findings at multiple spinal levels in this study may limit the discernability for potential targets for intervention based on imaging findings alone.

### 4.3 Limitations

Several limitations should be considered when interpreting the present study. First, imaging was coded categorically as present or absent without taking into account the severity of findings. The severity of neural foraminal stenosis, tendinopathy, or degenerative change has the potential to meaningfully influence the presence of symptoms. This was limited by the consistency with which radiology reports commented on severity. Additionally, descriptions of imaging findings were qualitative without standardization of language. Inter-reader reliability of MRI can be variable with one study reporting inter-rater agreement of neural foraminal stenosis on cervical MRI of 73% [23].

Second, as an exploratory study, more robust statistical analyses were limited by the data available from chart review. For example, though we collected data over a 10-year period, only 27% of patients with shoulder MRIs had a shoulder MRI of the asymptomatic side within the study period. As this data was not missing at random (i.e. imaging of the asymptomatic side is less likely to be ordered), analysis of the association of symptom laterality with shoulder imaging findings was not performed.

Third, MRIs up to 10 years after the initial clinical presentation of scapular pain were included in the study, and while the third quartile was 31 months for time from initial orthopedic visit to first MRI, there may be an increasing number of incidental findings with increasing age [15], [16], [18], [19], [20].

## 5 Conclusion

MRI findings in patients with scapular pain overlap with findings that occur in the general population with increasing age, and this highlights the complexity of evaluating this patient population even when imaging is ordered in their diagnostic workup. Future prospective studies with standardized MRI ordering and interpretations are needed to determine the clinical utility of imaging in patients who present with scapular pain.

## Author disclosures

a. Competing Interests: The authors declare that they have no known competing financial interests or personal relationships that could have appeared to influence the work reported in this paper.
b. Funding or Grants: This research did not receive any specific grant from funding agencies in the public, commercial, or not-for-profit sectors.
c. Financial benefits to authors: There were no financial benefits received for this study.
d. Submission declaration or verification: This work has not been previously published.

## Data availability statement

The datasets generated during and/or analyzed during the current study are available from the corresponding author on reasonable request.
